# Supplementary material for: Epigenetic clocks moderate the impact of marital status transitions on health in older adults
Source: PLoS One. 2026 May 13;21(5):e0327077. doi: 10.1371/journal.pone.0327077 (PMC13170869; doi:10.1371/journal.pone.0327077)
Supplement: S1 Table — (PDF) [file pone.0327077.s001.pdf]

S1 Table. Ordinary Least Squares Models Using Marital Status, PGSs, and Social Factors to Predict Epigenetic Clocks (HRS)

	Model 1	Model 2	Model 3	Model 4	Model 5	Model 6	Model 7	Model 8	Model 9	Model 10	Model 11	Model 12	Model 13
VARIABLES	Horvath 1	Hannum	Levine	Horvath 2	Lin	Weidner	Vidal-Bralo	EpiTOC	Zhang	Bocklandt	Garagnani	GrimAge	DunedinPACE
2014 Marital Status (Ref. = Married/Partnered)													
Separated/Divorced	0.269	-0.584	-0.707	-0.191	-0.251	-1.942*	0.075	0.001	-0.017	-0.004	-0.001	0.211	0.002
	(0.490)	(0.404)	(0.557)	(0.360)	(0.557)	(0.759)	(0.397)	(0.001)	(0.031)	(0.006)	(0.004)	(0.315)	(0.007)
Widowed	-0.705	0.043	0.396	-0.138	0.448	0.799	0.529	-0.001	0.061+	-0.007	-0.004	0.488	0.016*
	(0.502)	(0.411)	(0.564)	(0.373)	(0.650)	(0.890)	(0.429)	(0.001)	(0.033)	(0.006)	(0.004)	(0.329)	(0.007)
Never Married	0.995	0.148	-0.487	0.129	1.668+	-0.669	0.621	-0.002	-0.033	-0.014	-0.004	0.577	0.015
	(0.815)	(0.659)	(1.175)	(0.498)	(0.977)	(1.450)	(0.792)	(0.002)	(0.064)	(0.010)	(0.007)	(0.600)	(0.013)
2014 Health Lifestyle													
Vigorous Physical Activity	-0.648+	-0.310	-0.574	-0.342	0.063	-0.441	-0.315	-0.001	-0.012	-0.000	-0.004	-0.576**	-0.011*
	(0.348)	(0.263)	(0.353)	(0.242)	(0.433)	(0.567)	(0.254)	(0.001)	(0.022)	(0.004)	(0.003)	(0.214)	(0.005)
Ever Drinks Any Alcohol	-0.765*	-0.266	0.391	-0.533*	-0.246	1.058+	-0.257	-0.001	-0.025	0.007*	0.000	-0.392+	-0.004
	(0.330)	(0.279)	(0.359)	(0.224)	(0.415)	(0.561)	(0.258)	(0.001)	(0.022)	(0.003)	(0.003)	(0.219)	(0.005)
Ever Smokes	0.122	-0.083	0.304	-0.102	-0.060	-0.104	0.095	-0.000	0.161***	-0.002	0.002	3.078***	0.043***
	(0.304)	(0.246)	(0.330)	(0.217)	(0.375)	(0.523)	(0.238)	(0.001)	(0.020)	(0.003)	(0.003)	(0.189)	(0.004)
Polygenic Scores													
Longevity PGS	-0.057	-0.175	-0.080	-0.173	-0.042	-0.455	-0.055	-0.001	0.001	-0.002	0.001	0.127	0.000
	(0.195)	(0.147)	(0.199)	(0.127)	(0.230)	(0.313)	(0.156)	(0.001)	(0.012)	(0.002)	(0.002)	(0.111)	(0.002)
NCEB PGS	-0.238	-0.045	0.483*	-0.128	0.151	-0.085	-0.063	-0.000	0.018	-0.000	-0.001	0.106	0.002
	(0.197)	(0.160)	(0.206)	(0.129)	(0.223)	(0.318)	(0.150)	(0.001)	(0.012)	(0.002)	(0.002)	(0.124)	(0.003)
AFB PGS	0.052	-0.003	-0.026	0.021	0.111	-0.445	-0.109	-0.000	-0.007	-0.001	0.000	-0.153	-0.000
	(0.175)	(0.143)	(0.183)	(0.118)	(0.216)	(0.302)	(0.132)	(0.001)	(0.011)	(0.002)	(0.002)	(0.108)	(0.002)
Socioeconomic Background													
Years of Education	-0.025	0.059	-0.081	-0.064	0.138	0.242+	0.028	-0.000	-0.008	-0.001	0.000	-0.220***	-0.003*
	(0.075)	(0.067)	(0.085)	(0.055)	(0.102)	(0.138)	(0.060)	(0.000)	(0.005)	(0.001)	(0.001)	(0.049)	(0.001)
Parental Years of Education	-0.082	-0.143**	-0.106+	-0.049	0.094	-0.140	-0.037	-0.001***	-0.010**	-0.001	-0.002***	-0.085*	-0.001
	(0.057)	(0.047)	(0.061)	(0.041)	(0.071)	(0.107)	(0.047)	(0.000)	(0.004)	(0.001)	(0.000)	(0.039)	(0.001)
2014 Total of All Assets	-0.009	-0.015	-0.022*	-0.005	-0.022	-0.018	-0.006	-0.000	-0.002**	0.000	0.000	-0.017*	-0.000
	(0.012)	(0.010)	(0.011)	(0.007)	(0.015)	(0.025)	(0.008)	(0.000)	(0.001)	(0.000)	(0.000)	(0.008)	(0.000)
2014 Retirement Status (Ref. = Not retired)													
Completely Retired	0.411	0.051	0.604	0.312	-0.193	-0.315	0.237	0.001	0.032	0.005	0.003	0.616*	0.000
	(0.405)	(0.319)	(0.464)	(0.288)	(0.487)	(0.681)	(0.299)	(0.001)	(0.029)	(0.005)	(0.004)	(0.273)	(0.006)
Partly Retired	0.596	0.030	0.874+	0.348	-0.325	0.350	0.314	0.001	0.011	0.002	0.002	0.380	0.001
	(0.457)	(0.379)	(0.520)	(0.329)	(0.572)	(0.797)	(0.360)	(0.001)	(0.032)	(0.005)	(0.004)	(0.328)	(0.007)
Question Irrelevant	-2.167*	-1.106	-1.375	-1.231+	-2.035	0.878	-0.140	-0.003	-0.003	0.004	-0.015+	0.417	0.009
	(0.884)	(0.739)	(0.880)	(0.661)	(1.293)	(1.932)	(0.615)	(0.002)	(0.070)	(0.011)	(0.008)	(0.611)	(0.014)
Demographic Characteristics													
Female	-1.055**	-2.055***	-1.406***	-1.142***	-1.291***	-1.167*	-1.953***	0.001	-0.201***	0.021***	0.005+	-3.244***	-0.018***
	(0.326)	(0.267)	(0.369)	(0.225)	(0.391)	(0.555)	(0.244)	(0.001)	(0.021)	(0.004)	(0.003)	(0.203)	(0.004)
2014 Age	0.695***	0.758***	0.705***	0.776***	0.799***	0.552***	0.346***	0.000***	0.012***	-0.003***	0.005***	0.658***	-0.000
	(0.043)	(0.036)	(0.049)	(0.029)	(0.058)	(0.080)	(0.036)	(0.000)	(0.003)	(0.000)	(0.000)	(0.027)	(0.001)
Cohort (Ref. = Old)													
Middle	1.018+	1.028*	-0.264	1.171**	0.519	-1.263	-0.332	-0.001	0.000	0.003	0.011*	-0.465	-0.006
	(0.571)	(0.476)	(0.612)	(0.381)	(0.704)	(0.942)	(0.448)	(0.002)	(0.036)	(0.006)	(0.005)	(0.361)	(0.008)
Young	-0.709	0.011	-0.398	0.170	-0.713	1.124	-0.246	-0.001	-0.003	0.004	0.012	-1.194*	-0.016
	(0.770)	(0.682)	(0.891)	(0.540)	(1.080)	(1.465)	(0.668)	(0.002)	(0.053)	(0.009)	(0.007)	(0.532)	(0.012)
2014 Family Size	0.308+	0.047	0.245	-0.083	0.592**	0.414	0.426**	0.000	0.008	-0.005*	-0.001	0.082	0.002
	(0.174)	(0.139)	(0.216)	(0.148)	(0.215)	(0.299)	(0.132)	(0.000)	(0.012)	(0.002)	(0.001)	(0.122)	(0.002)
2014 Number of Living Siblings	0.131	-0.033	-0.138	-0.035	0.062	0.135	0.045	0.000	0.004	-0.001	-0.000	-0.076	-0.001
	(0.084)	(0.065)	(0.089)	(0.058)	(0.102)	(0.142)	(0.063)	(0.000)	(0.005)	(0.001)	(0.001)	(0.055)	(0.001)
Religious Affiliation (Ref. = Protestant)													
Catholics	-0.164	-0.640+	-0.428	0.142	0.077	-0.238	-0.291	-0.000	-0.060*	-0.001	0.001	0.012	-0.003
	(0.419)	(0.350)	(0.423)	(0.291)	(0.492)	(0.715)	(0.309)	(0.001)	(0.027)	(0.004)	(0.004)	(0.266)	(0.006)
None	0.127	-0.332	0.048	-0.030	-0.498	0.524	0.335	0.000	0.004	-0.003	-0.008*	0.065	-0.002
	(0.520)	(0.380)	(0.579)	(0.349)	(0.628)	(0.850)	(0.407)	(0.001)	(0.030)	(0.006)	(0.004)	(0.322)	(0.007)
Other	0.420	1.040	0.771	-0.535	0.660	1.498	-0.244	-0.001	0.024	-0.007	0.005	0.976+	-0.004
	(0.980)	(1.036)	(1.363)	(0.659)	(1.174)	(2.007)	(0.779)	(0.003)	(0.070)	(0.011)	(0.010)	(0.564)	(0.013)
Population Stratification													
PC1	38.807*	18.667	20.682	18.922	17.085	-45.714	-11.862	0.027	0.434	0.072	0.234	-24.885*	-0.264
	(19.026)	(18.923)	(22.520)	(14.094)	(21.011)	(31.710)	(15.473)	(0.058)	(1.398)	(0.216)	(0.178)	(11.978)	(0.266)
PC2	11.323	-9.943	-7.477	9.235	-17.424	-20.886	-3.243	-0.064	-1.449	0.216	0.126	-1.919	0.145
	(16.494)	(12.887)	(17.568)	(11.592)	(19.922)	(30.003)	(13.226)	(0.050)	(1.053)	(0.177)	(0.130)	(10.187)	(0.227)
PC3	4.943	-4.025	-24.394	-16.107	-16.576	8.323	-4.076	-0.010	1.545	0.099	0.027	16.243	0.382+
	(17.321)	(14.021)	(18.322)	(11.894)	(22.833)	(28.363)	(13.088)	(0.046)	(1.156)	(0.187)	(0.146)	(10.286)	(0.225)
PC4	-22.842	-15.763	-1.900	-18.353	11.742	52.317+	0.569	0.007	0.802	0.165	0.136	-0.133	0.202

	(17.096)	(13.351)	(18.627)	(11.628)	(20.992)	(30.706)	(13.971)	(0.041)	(1.177)	(0.194)	(0.144)	(10.958)	(0.249)
PC5	-5.905	9.089	-8.447	1.296	-5.210	-53.613	-7.414	-0.093	-0.844	-0.508+	0.208	13.038	0.195
	(24.455)	(22.517)	(27.358)	(17.187)	(28.778)	(40.939)	(20.573)	(0.067)	(1.632)	(0.266)	(0.222)	(13.952)	(0.315)
PC6	-45.343*	-6.815	-26.332	-18.724+	-42.671*	21.097	9.605	-0.019	0.787	0.377*	0.013	3.361	-0.083
	(18.353)	(13.909)	(17.423)	(11.260)	(20.019)	(27.096)	(13.153)	(0.047)	(1.048)	(0.179)	(0.149)	(10.765)	(0.237)
PC7	-24.521	-14.915	-34.023+	-2.992	-28.179	1.423	-7.828	0.004	0.261	-0.026	-0.009	4.711	0.187
	(17.449)	(13.458)	(19.285)	(11.495)	(20.480)	(28.641)	(14.742)	(0.044)	(1.099)	(0.190)	(0.151)	(10.946)	(0.237)
PC8	-23.338	-11.405	-1.748	0.893	-26.476	11.356	-18.138	-0.080+	-0.547	0.047	0.117	14.113	0.255
	(16.649)	(12.933)	(17.862)	(11.945)	(19.484)	(29.110)	(12.619)	(0.045)	(1.167)	(0.187)	(0.156)	(11.122)	(0.244)
PC9	13.759	25.581+	3.865	17.885+	21.909	-39.141	13.026	0.062	1.793+	-0.181	0.036	4.263	0.036
	(16.419)	(13.532)	(17.173)	(10.850)	(19.757)	(28.066)	(13.218)	(0.045)	(1.088)	(0.170)	(0.137)	(10.051)	(0.218)
PC10	-3.749	-9.254	-2.725	3.657	-10.995	-19.454	-10.050	0.058	-0.550	0.062	-0.081	-10.823	-0.190
	(19.831)	(14.763)	(18.479)	(12.671)	(21.234)	(30.186)	(14.671)	(0.050)	(1.118)	(0.191)	(0.161)	(10.239)	(0.230)
Constant	20.786***	6.169*	12.378**	19.923***	1.877	26.887***	41.055***	0.047***	-1.632***	0.615***	0.358***	28.076***	1.137***
	(3.656)	(2.954)	(3.967)	(2.440)	(5.024)	(6.629)	(2.939)	(0.010)	(0.232)	(0.039)	(0.031)	(2.313)	(0.050)
Observations	1,654	1,654	1,654	1,654	1,654	1,654	1,654	1,654	1,654	1,654	1,654	1,654	1,654
Adjusted R-squared	0.537	0.663	0.515	0.741	0.479	0.162	0.327	0.0723	0.187	0.164	0.445	0.775	0.0866

Standard errors (in parentheses) are bias-corrected and accelerated (BCa) bootstrap standard errors based on 1,000 replications.
*** p<0.001, ** p<0.01, * p<0.05, + p<0.1
