# Supplementary material for: Epigenetic clocks moderate the impact of marital status transitions on health in older adults
Source: PLoS One. 2026 May 13;21(5):e0327077. doi: 10.1371/journal.pone.0327077 (PMC13170869; doi:10.1371/journal.pone.0327077)
Supplement: S2 Table — (PDF) [file pone.0327077.s002.pdf]

S2 Table. Ordinary Least Squares Models Using Marital Status, PGSs, and Social Factors to Predict Epigenetic Clocks for Males and Females (HRS)

**a. Males (N=598)**	Model 1	Model 2	Model 3	Model 4	Model 5	Model 6	Model 7	Model 8	Model 9	Model 10	Model 11	Model 12	Model 13
VARIABLES	Horvath 1	Hannum	Levine	Horvath 2	Lin	Weidner	Vidal-Bralo	EpiTOC (Yang)	Zhang	Bocklandt	Garagnani	GrimAge	DunedinPACE
2014 Marital Status (Ref. = Married/Partnered)													
Separated/Divorced	0.737[#]	-1.079[#]	-1.249[#]	-0.361[#]	-0.133[#]	-1.335[#]	-0.082[#]	-0.001[#]	-0.085+[#]	-0.013[#]	0.005[#]	-0.180[#]	0.737[#]
	(0.765)	(0.704)	(0.918)	(0.638)	(0.927)	(1.407)	(0.677)	(0.002)	(0.052)	(0.010)	(0.007)	(0.607)	(0.765)
Widowed	-0.436	-0.314	2.079	-0.507	0.855	1.466	2.340+	-0.000	0.048	-0.038*	-0.013	0.008	-0.436
	(1.405)	(0.946)	(1.581)	(1.299)	(1.713)	(2.508)	(1.206)	(0.003)	(0.091)	(0.015)	(0.010)	(1.018)	(1.405)
Never Married	0.791	0.823	-1.941	-0.528	-0.190	-2.353	-0.277	0.001	0.024	-0.004	-0.008	1.031	0.791
	(1.146)	(0.928)	(1.408)	(0.715)	(1.497)	(2.281)	(1.181)	(0.003)	(0.101)	(0.019)	(0.010)	(0.982)	(1.146)
Adjusted R-squared	0.555	0.633	0.493	0.714	0.459	0.106	0.295	0.0609	0.124	0.135	0.448	0.734	0.555

**b. Females (N=851)**	Model 1	Model 2	Model 3	Model 4	Model 5	Model 6	Model 7	Model 8	Model 9	Model 10	Model 11	Model 12	Model 13
VARIABLES	Horvath 1	Hannum	Levine	Horvath 2	Lin	Weidner	Vidal-Bralo	EpiTOC (Yang)	Zhang	Bocklandt	Garagnani	GrimAge	DunedinPACE
2014 Marital Status (Ref. = Married/Partnered)													
Separated/Divorced	0.023[#]	-0.307[#]	-0.559[#]	-0.138[#]	-0.225[#]	-1.986*[#]	0.202[#]	0.002[#]	0.013[#]	-0.001[#]	-0.004[#]	0.328[#]	0.007[#]
	(0.643)	(0.482)	(0.692)	(0.447)	(0.759)	(0.965)	(0.543)	(0.002)	(0.042)	(0.007)	(0.006)	(0.416)	(0.009)
Widowed	-0.555	0.286	-0.030	0.126	0.383	0.169	0.180	-0.001	0.054	-0.003	-0.001	0.556	0.018*
	(0.599)	(0.459)	(0.635)	(0.417)	(0.745)	(0.973)	(0.469)	(0.002)	(0.037)	(0.006)	(0.005)	(0.360)	(0.009)
Never Married	1.384	-0.165	0.935	0.664	3.107*	0.655	1.746	-0.004*	-0.067	-0.027*	-0.001	0.093	0.017
	(1.306)	(1.013)	(1.780)	(0.797)	(1.283)	(1.937)	(1.103)	(0.002)	(0.083)	(0.012)	(0.010)	(0.803)	(0.019)
Adjusted R-squared	0.522	0.675	0.521	0.758	0.487	0.195	0.320	0.0799	0.148	0.155	0.439	0.785	0.0836

Standard errors (in parentheses) are bias-corrected and accelerated (BCa) bootstrap standard errors based on 1,000 replications.

# indicates a statistically significant male–female difference based on pooled OLS models with gender interactions (p<0.05).

*** p<0.001, ** p<0.01, * p<0.05, + p<0.1

Notes: The following variables are controlled in the models: health lifestyles in 2014, polygenic scores (longevity PGS, number of children ever born PGS, and age at first birth PGS), social support in 2014, educational attainment, parental education, total of all assets in 2014, retirement status in 2014, chronological age at 2014, cohort, family size in 2014, number of living siblings in 2014, religious affiliation, and population stratification.
