# Supplementary material for: Epigenetic clocks moderate the impact of marital status transitions on health in older adults
Source: PLoS One. 2026 May 13;21(5):e0327077. doi: 10.1371/journal.pone.0327077 (PMC13170869; doi:10.1371/journal.pone.0327077)
Supplement: S3 Table — (PDF) [file pone.0327077.s003.pdf]

S3 Table. Ordinary Least Squares Models Using Epigenetic Clocks and Marital Status Change to Predict CESD Score in 2020 (HRS)

VARIABLES	Model 1<br>2020<br>CESD<br>Horvath 1	Model 2<br>2020<br>CESD<br>Hannum	Model 3<br>2020<br>CESD<br>Levine	Model 4<br>2020<br>CESD<br>Horvath 2	Model 5<br>2020<br>CESD<br>Lin	Model 6<br>2020<br>CESD<br>Weidner	Model 7<br>2020<br>CESD<br>Vidal-Bralo	Model 8<br>2020<br>CESD<br>EpiTOC	Model 9<br>2020<br>CESD<br>Zhang	Model 10<br>2020<br>CESD<br>Bocklandt	Model 11<br>2020<br>CESD<br>Garagnani	Model 12<br>2020<br>CESD<br>GrimAge	Model 13<br>2020 CESD<br>DunedinPoAm
Epigenetic Clock	0.008	0.016*	0.009	0.009	0.007	-0.004	-0.001	-0.917	0.074	-0.678	1.413+	0.038**[b,f]	1.037+
	(0.007)	(0.008)	(0.007)	(0.010)	(0.006)	(0.005)	(0.009)	(2.185)	(0.104)	(0.650)	(0.805)	(0.012)	(0.539)
2016-20 Marital Status Change	0.620**[b,f]	0.614**[b,f]	0.609**[b,f]	0.619**[b,f]	0.613**[b,f]	0.617**[b,f]	0.616**[b,f]	0.615**[b,f]	0.614**[b,f]	0.619**[b,f]	0.623**[b,f]	0.609**[b,f]	0.620**[b,f]
	(0.191)	(0.191)	(0.190)	(0.191)	(0.191)	(0.191)	(0.192)	(0.191)	(0.191)	(0.191)	(0.191)	(0.191)	(0.191)
2016 Marital Status (Ref. = Married/Partnered)													
Separated/Divorced	0.300+	0.317+	0.308+	0.307+	0.304+	0.294	0.303+	0.303+	0.306+	0.299	0.309+	0.294	0.300+
	(0.182)	(0.182)	(0.182)	(0.182)	(0.182)	(0.183)	(0.182)	(0.182)	(0.182)	(0.183)	(0.183)	(0.181)	(0.181)
Widowed	0.260	0.254	0.250	0.257	0.251	0.258	0.254	0.255	0.249	0.251	0.262	0.233	0.236
	(0.162)	(0.162)	(0.162)	(0.162)	(0.162)	(0.162)	(0.163)	(0.162)	(0.163)	(0.162)	(0.162)	(0.161)	(0.163)
Never Married	0.357	0.359	0.372	0.365	0.358	0.364	0.368	0.365	0.369	0.362	0.368	0.345	0.349
	(0.294)	(0.293)	(0.294)	(0.294)	(0.295)	(0.294)	(0.293)	(0.294)	(0.293)	(0.293)	(0.294)	(0.289)	(0.297)
Health Lifestyle													
Vigorous Physical Activity	-0.444***	-0.444***	-0.439***	-0.442***	-0.447***	-0.448***	-0.445***	-0.446***	-0.440***	-0.445***	-0.447***	-0.411***	-0.434***
	(0.097)	(0.097)	(0.098)	(0.097)	(0.096)	(0.097)	(0.097)	(0.097)	(0.098)	(0.097)	(0.097)	(0.097)	(0.097)
Ever Drinks Any Alcohol	-0.062	-0.061	-0.067	-0.060	-0.064	-0.062	-0.065	-0.066	-0.062	-0.060	-0.068	-0.051	-0.061
	(0.106)	(0.106)	(0.106)	(0.106)	(0.106)	(0.106)	(0.106)	(0.106)	(0.106)	(0.106)	(0.106)	(0.105)	(0.106)
Ever Smokes	-0.044	-0.042	-0.045	-0.042	-0.042	-0.043	-0.042	-0.043	-0.054	-0.044	-0.045	-0.159	-0.087
	(0.095)	(0.095)	(0.095)	(0.095)	(0.095)	(0.095)	(0.095)	(0.095)	(0.095)	(0.095)	(0.095)	(0.099)	(0.098)
Polygenic Scores													
Longevity PGS	-0.095+	-0.092+	-0.094+	-0.094+	-0.095+	-0.097+	-0.095+	-0.096+	-0.095+	-0.096+	-0.096+	-0.100+	-0.096+
	(0.056)	(0.056)	(0.056)	(0.056)	(0.056)	(0.056)	(0.056)	(0.056)	(0.056)	(0.056)	(0.056)	(0.056)	(0.056)
Depressive Symptoms PGS	0.088+	0.086+	0.088+	0.090+	0.087+	0.089+	0.087+	0.087+	0.086+	0.086+	0.087+	0.085+	0.088+
	(0.049)	(0.049)	(0.049)	(0.049)	(0.049)	(0.049)	(0.049)	(0.049)	(0.049)	(0.049)	(0.049)	(0.049)	(0.049)
Socioeconomic Background													
Years of Education	-0.032	-0.034	-0.032	-0.032	-0.034	-0.032	-0.033	-0.033	-0.032	-0.033	-0.033	-0.025	-0.030
	(0.023)	(0.023)	(0.023)	(0.023)	(0.023)	(0.023)	(0.023)	(0.023)	(0.023)	(0.023)	(0.023)	(0.023)	(0.023)
Parental Years of Education	-0.013	-0.012	-0.013	-0.014	-0.015	-0.015	-0.014	-0.015	-0.013	-0.015	-0.011	-0.011	-0.013
	(0.018)	(0.018)	(0.018)	(0.018)	(0.018)	(0.018)	(0.018)	(0.018)	(0.018)	(0.018)	(0.018)	(0.018)	(0.018)
2016 Total of All Assets	-0.008*	-0.008*	-0.008*	-0.008*	-0.008*	-0.008*	-0.008*	-0.008*	-0.008*	-0.008*	-0.008*	-0.007*	-0.008*
	(0.004)	(0.004)	(0.004)	(0.004)	(0.004)	(0.004)	(0.004)	(0.004)	(0.004)	(0.004)	(0.004)	(0.003)	(0.004)
2016 Retirement Status (Ref. = Not retired)													
Completely Retired	0.533***	0.533***	0.534***	0.534***	0.539***	0.538***	0.541***	0.542***	0.538***	0.543***	0.537***	0.511***	0.530***
	(0.145)	(0.144)	(0.145)	(0.145)	(0.145)	(0.144)	(0.145)	(0.144)	(0.145)	(0.144)	(0.145)	(0.144)	(0.144)
Partly Retired	0.364*	0.361*	0.365*	0.362*	0.366*	0.363*	0.364*	0.362*	0.364*	0.364*	0.357*	0.359*	0.364*
	(0.161)	(0.160)	(0.161)	(0.161)	(0.161)	(0.161)	(0.161)	(0.161)	(0.161)	(0.160)	(0.161)	(0.160)	(0.161)
Question Irrelevant	0.180	0.179	0.190	0.183	0.186	0.180	0.186	0.183	0.185	0.187	0.207	0.185	0.169
	(0.346)	(0.350)	(0.346)	(0.347)	(0.345)	(0.345)	(0.346)	(0.346)	(0.346)	(0.343)	(0.345)	(0.344)	(0.349)
Demographic Characteristics													
Female	0.216*	0.241*	0.221*	0.218*	0.216*	0.202+	0.205+	0.207*	0.222*	0.221*	0.198+	0.335**	0.227*
	(0.106)	(0.108)	(0.106)	(0.107)	(0.106)	(0.105)	(0.108)	(0.105)	(0.108)	(0.107)	(0.105)	(0.110)	(0.106)
2020 Age	-0.014	-0.021	-0.015	-0.016	-0.014	-0.007	-0.009	-0.009	-0.010	-0.011	-0.017	-0.034*	-0.008
	(0.014)	(0.015)	(0.014)	(0.015)	(0.014)	(0.013)	(0.013)	(0.013)	(0.013)	(0.013)	(0.014)	(0.015)	(0.013)
Cohort (Ref. = Old)													
Middle	-0.105	-0.114	-0.096	-0.108	-0.101	-0.102	-0.098	-0.099	-0.098	-0.096	-0.114	-0.080	-0.091
	(0.162)	(0.161)	(0.161)	(0.161)	(0.161)	(0.161)	(0.161)	(0.161)	(0.161)	(0.161)	(0.161)	(0.160)	(0.161)
Young	0.267	0.261	0.266	0.261	0.266	0.266	0.261	0.260	0.261	0.264	0.244	0.306	0.276
	(0.244)	(0.244)	(0.244)	(0.244)	(0.245)	(0.244)	(0.244)	(0.244)	(0.245)	(0.245)	(0.245)	(0.244)	(0.244)
2016 Family Size	-0.018	-0.014	-0.016	-0.014	-0.018	-0.015	-0.014	-0.014	-0.014	-0.016	-0.014	-0.016	-0.017
	(0.062)	(0.062)	(0.062)	(0.062)	(0.062)	(0.062)	(0.062)	(0.062)	(0.062)	(0.062)	(0.062)	(0.062)	(0.062)
2016 Number of Living Siblings	-0.042	-0.040	-0.040	-0.041	-0.041	-0.040	-0.041	-0.041	-0.041	-0.042	-0.041	-0.038	-0.040
	(0.026)	(0.026)	(0.026)	(0.026)	(0.026)	(0.026)	(0.026)	(0.026)	(0.026)	(0.026)	(0.026)	(0.026)	(0.026)
Religious Affiliation (Ref. = Protestant)													
Catholics	-0.206	-0.198	-0.205	-0.209	-0.209	-0.209	-0.208	-0.208	-0.204	-0.209	-0.208	-0.209+	-0.204
	(0.128)	(0.128)	(0.127)	(0.127)	(0.128)	(0.128)	(0.127)	(0.127)	(0.127)	(0.128)	(0.128)	(0.126)	(0.127)
None	0.284+	0.290+	0.284+	0.285+	0.288+	0.287+	0.285+	0.285+	0.285+	0.283+	0.297+	0.282+	0.287+
	(0.166)	(0.166)	(0.166)	(0.166)	(0.166)	(0.166)	(0.166)	(0.166)	(0.166)	(0.166)	(0.166)	(0.166)	(0.166)
Other	0.102	0.088	0.099	0.111	0.101	0.109	0.104	0.103	0.102	0.100	0.097	0.071	0.108
	(0.459)	(0.459)	(0.460)	(0.458)	(0.458)	(0.459)	(0.458)	(0.458)	(0.458)	(0.458)	(0.460)	(0.462)	(0.461)
Population Stratification													
PC1	1.958	1.983	2.137	2.068	2.161	2.079	2.260	2.294	2.272	2.314	1.920	3.357	2.598
	(6.782)	(6.779)	(6.810)	(6.786)	(6.783)	(6.782)	(6.801)	(6.797)	(6.787)	(6.788)	(6.839)	(6.851)	(6.828)
PC2	-3.692	-3.499	-3.642	-3.704	-3.546	-3.705	-3.639	-3.701	-3.567	-3.496	-3.797	-3.681	-3.848
	(5.462)	(5.478)	(5.471)	(5.451)	(5.465)	(5.436)	(5.446)	(5.471)	(5.459)	(5.467)	(5.425)	(5.444)	(5.450)
PC3	-7.180	-6.993	-6.923	-7.044	-7.002	-7.158	-7.113	-7.123	-7.177	-7.037	-7.138	-7.564	-7.554
	(5.263)	(5.258)	(5.254)	(5.264)	(5.261)	(5.258)	(5.253)	(5.254)	(5.262)	(5.268)	(5.286)	(5.274)	(5.260)
PC4	-5.285	-5.168	-5.362	-5.235	-5.484	-5.235	-5.414	-5.400	-5.452	-5.293	-5.638	-5.347	-5.618
	(5.530)	(5.523)	(5.518)	(5.540)	(5.528)	(5.524)	(5.527)	(5.522)	(5.516)	(5.526)	(5.494)	(5.485)	(5.511)
PC5	-15.675*	-15.943*	-15.874*	-15.728*	-15.760*	-15.944*	-15.797*	-15.879*	-15.799*	-16.089*	-16.010*	-16.607*	-16.099*

	(7.735)	(7.740)	(7.771)	(7.737)	(7.735)	(7.762)	(7.760)	(7.755)	(7.753)	(7.780)	(7.766)	(7.776)	(7.805)
PC6	-6.685	-6.910	-6.845	-6.875	-6.754	-6.973	-7.030	-7.057	-7.101	-6.764	-7.044	-7.191	-6.962
	(5.086)	(5.055)	(5.050)	(5.067)	(5.072)	(5.056)	(5.047)	(5.046)	(5.062)	(5.048)	(5.058)	(5.072)	(5.068)
PC7	-6.335	-6.297	-6.237	-6.516	-6.357	-6.549	-6.557	-6.546	-6.567	-6.560	-6.547	-6.642	-6.690
	(4.965)	(4.993)	(5.011)	(4.972)	(4.968)	(4.969)	(4.982)	(4.982)	(4.991)	(4.978)	(4.992)	(4.999)	(4.991)
PC8	-5.240	-5.227	-5.428	-5.405	-5.257	-5.392	-5.467	-5.530	-5.416	-5.419	-5.578	-5.890	-5.718
	(5.480)	(5.486)	(5.477)	(5.489)	(5.471)	(5.496)	(5.494)	(5.503)	(5.488)	(5.491)	(5.491)	(5.516)	(5.493)
PC9	2.267	1.965	2.373	2.212	2.250	2.234	2.390	2.436	2.261	2.267	2.301	2.298	2.349
	(5.181)	(5.161)	(5.168)	(5.180)	(5.164)	(5.192)	(5.181)	(5.191)	(5.182)	(5.177)	(5.163)	(5.187)	(5.194)
PC10	1.442	1.601	1.446	1.367	1.486	1.339	1.417	1.482	1.492	1.466	1.550	1.864	1.616
	(5.096)	(5.107)	(5.091)	(5.081)	(5.098)	(5.112)	(5.099)	(5.086)	(5.105)	(5.094)	(5.078)	(5.095)	(5.097)
Constant	2.139+	2.227+	2.182+	2.117+	2.278+	2.357*	2.299+	2.305+	2.386*	2.691*	1.823	1.336	1.076
	(1.169)	(1.170)	(1.165)	(1.173)	(1.172)	(1.177)	(1.218)	(1.181)	(1.179)	(1.225)	(1.186)	(1.181)	(1.259)
Observations	1,654	1,654	1,654	1,654	1,654	1,654	1,654	1,654	1,654	1,654	1,654	1,654	1,654
Adjusted R-squared	0.0557	0.0569	0.0560	0.0555	0.0558	0.0555	0.0551	0.0552	0.0554	0.0557	0.0567	0.0610	0.0572

Standard errors (in parentheses) are bias-corrected and accelerated (BCa) bootstrap standard errors based on 1,000 replications.
*** p<0.001, ** p<0.01, * p<0.05, + p<0.1, b: significant after Bonferroni correction, f: significant after FDR correction
