# Supplementary material for: Epigenetic clocks moderate the impact of marital status transitions on health in older adults
Source: PLoS One. 2026 May 13;21(5):e0327077. doi: 10.1371/journal.pone.0327077 (PMC13170869; doi:10.1371/journal.pone.0327077)
Supplement: S8 Table — (PDF) [file pone.0327077.s008.pdf]

S8 Table. Cox Proportional Hazards Regression Models Using Interaction Term Between Epigenetic Clocks and Marital Status Change to Predict Mortality Risk for Males and Females (HRS)

a. Males (N=865)	Model 1	Model 2	Model 3	Model 4	Model 5	Model 6	Model 7	Model 8	Model 9	Model 10	Model 11	Model 12	Model 13
VARIABLES	Horvath 1	Hannum	Levine	Horvath 2	Lin	Weidner	Vidal-Bralo	EpiTOC	Zhang	Bocklandt	Garagnani	GrimAge	DunedinPACE
Models with Main Effects													
Epigenetic Clock	0.021	0.042**[f]	0.039**[b,f]	0.032	0.021*	0.012+	0.018	-3.592	0.976***[b,f]	-1.531	2.791+	0.112***[b,f]	4.061***[b,f]
	(0.015)	(0.015)	(0.012)	(0.021)	(0.010)	(0.007)	(0.014)	(4.457)	(0.222)	(1.185)	(1.661)	(0.018)	(0.938)
2012-16 Marital Status Change	0.011	-0.110	0.055	-0.025	0.033	-0.001	0.008	0.056	-0.156	0.083	-0.024	0.118	0.157
	(0.326)	(0.337)	(0.324)	(0.326)	(0.330)	(0.332)	(0.324)	(0.325)	(0.355)	(0.322)	(0.329)	(0.311)	(0.313)
2016 Marital Status (Ref. = Married/Partnered)													
Separated/Divorced	0.525	0.614+	0.544+	0.531+	0.571+	0.569+	0.555+	0.514	0.706*[#]	0.497	0.550+	0.552+	0.533
	(0.320)	(0.322)	(0.323)	(0.322)	(0.327)	(0.333)	(0.327)	(0.325)	(0.327)	(0.330)	(0.323)	(0.323)	(0.330)
Widowed	0.583*	0.627*	0.481+	0.567+	0.545+	0.529+	0.562+	0.600*	0.533+	0.527+	0.618*	0.543+[#]	0.558*[#]
	(0.287)	(0.292)	(0.291)	(0.290)	(0.292)	(0.296)	(0.289)	(0.285)	(0.292)	(0.292)	(0.288)	(0.280)	(0.274)
Never Married	0.406	0.343	0.478	0.400	0.402	0.410	0.434	0.411	0.216	0.403	0.367	0.406	0.479
	(2.975)	(2.833)	(3.061)	(3.103)	(3.083)	(2.971)	(3.031)	(3.010)	(3.149)	(2.980)	(3.042)	(3.186)	(3.035)
Log-Likelihood	-1123	-1119	-1117	-1122	-1121	-1122	-1123	-1124	-1111	-1123	-1122	-1104	-1112
chi2	169.7	182.9	172.7	171.3	179.1	165.7	164.2	162.5	172.0	166.7	171.3	211.5	187.0
Models with Interaction Effects													
Epigenetic Clock	0.028	0.050**[b,f]	0.046***[b,f]	0.042+	0.030**[f]	0.016*	0.022	-1.821	1.231***[b,f#]	-1.331	3.377+	0.118***[b,f]	4.088***[b,f]
	(0.017)	(0.016)	(0.013)	(0.022)	(0.011)	(0.008)	(0.017)	(4.519)	(0.239)	(1.272)	(1.761)	(0.019)	(1.012)
2012-16 Marital Status Change	2.827	4.134*	2.917	4.424+	1.903	1.731	1.260	0.839	-1.072*[#]	0.546	4.440	6.237*	0.351
	(2.174)	(1.791)	(1.884)	(2.490)	(1.477)	(1.741)	(2.616)	(1.002)	(0.499)	(1.195)	(3.033)	(2.435)	(2.911)
Epigenetic Clock*2012-16 Marital Status Change	-0.038	-0.065*	-0.043[#]	-0.057+	-0.027	-0.023	-0.018	-10.315	-1.520**[b,f#]	-1.319	-5.758	-0.079*	-0.176
	(0.029)	(0.028)	(0.029)	(0.032)	(0.021)	(0.023)	(0.036)	(13.500)	(0.521)	(3.511)	(3.945)	(0.032)	(2.622)
Log-Likelihood	-1122	-1115	-1116	-1120	-1120	-1121	-1123	-1123	-1106	-1123	-1120	-1100	-1112
chi2	169.1	183.2	169.5	178.6	176.3	161.3	160.0	159.1	178.2	165.4	173.1	213.0	182.8
**b. Females (N=1,166)**	Model 1	Model 2	Model 3	Model 4	Model 5	Model 6	Model 7	Model 8	Model 9	Model 10	Model 11	Model 12	Model 13
VARIABLES	Horvath 1	Hannum	Levine	Horvath 2	Lin	Weidner	Vidal-Bralo	EpiTOC	Zhang	Bocklandt	Garagnani	GrimAge	DunedinPACE
Models with Main Effects													
Epigenetic Clock	0.001	0.017	0.034***[b,f]	0.008	0.003	0.006	0.038**[b,f]	0.543	0.840***[b,f]	-1.766+	-0.267	0.112***[b,f]	2.110*
	(0.011)	(0.013)	(0.010)	(0.017)	(0.009)	(0.006)	(0.013)	(3.144)	(0.169)	(1.006)	(1.231)	(0.019)	(0.902)
2012-16 Marital Status Change	0.441*	0.425*	0.426*	0.448*	0.439*	0.449*	0.456*	0.440*	0.388+	0.424*	0.441*	0.438*	0.426*
	(0.209)	(0.210)	(0.209)	(0.210)	(0.210)	(0.209)	(0.213)	(0.211)	(0.209)	(0.213)	(0.210)	(0.211)	(0.214)
2016 Marital Status (Ref. = Married/Partnered)													
Separated/Divorced	-0.168	-0.129	-0.093	-0.161	-0.169	-0.154	-0.163	-0.167	-0.121[#]	-0.202	-0.176	-0.110	-0.166
	(0.259)	(0.257)	(0.253)	(0.258)	(0.259)	(0.257)	(0.257)	(0.260)	(0.263)	(0.263)	(0.263)	(0.259)	(0.257)
Widowed	0.013	0.024	0.002	0.010	0.013	0.011	0.008	0.017	-0.013	-0.018	0.015	-0.023[#]	-0.034[#]
	(0.205)	(0.206)	(0.209)	(0.206)	(0.205)	(0.206)	(0.206)	(0.207)	(0.207)	(0.208)	(0.206)	(0.203)	(0.209)
Never Married	0.504	0.488	0.349	0.503	0.504	0.496	0.485	0.511	0.364	0.463	0.501	0.284	0.383
	(3.249)	(3.143)	(3.177)	(3.091)	(3.172)	(3.248)	(3.149)	(3.251)	(3.242)	(3.247)	(2.959)	(3.018)	(2.970)
Log-Likelihood	-1340	-1339	-1333	-1340	-1340	-1339	-1335	-1340	-1326	-1338	-1340	-1321	-1336
chi2	192.9	195.9	219.2	196.5	188.2	193.1	195.7	190.0	210.9	189.9	193.8	216.0	181.4
Models with Interaction Effects													
Epigenetic Clock	0.004	0.019	0.032**[b,f]	0.011	0.004	0.005	0.038**[f]	0.311	0.689***[b,f#]	-2.555*[f]	0.466	0.118***[b,f]	2.173*
	(0.012)	(0.014)	(0.010)	(0.018)	(0.009)	(0.007)	(0.014)	(3.531)	(0.180)	(1.081)	(1.336)	(0.020)	(0.978)
2012-16 Marital Status Change	1.739	1.636	-0.283	1.560	0.846	0.215	0.268	0.370	1.205**[b,f#]	-0.920	2.829	2.251	0.677
	(1.757)	(1.459)	(1.319)	(2.065)	(1.353)	(1.251)	(2.144)	(0.632)	(0.408)	(0.933)	(2.100)	(2.133)	(2.306)
Epigenetic Clock*2012-16 Marital Status Change	-0.018	-0.019	0.011[#]	-0.014	-0.006	0.003	0.003	0.941	1.090*[#]	3.810	-3.099	-0.024	-0.228
	(0.024)	(0.023)	(0.019)	(0.027)	(0.020)	(0.017)	(0.032)	(8.334)	(0.502)	(2.560)	(2.750)	(0.028)	(2.104)
Log-Likelihood	-1339	-1338	-1333	-1339	-1340	-1339	-1335	-1340	-1323	-1336	-1339	-1320	-1336
chi2	195.6	197.0	217.8	195.2	189.6	192.3	197.6	190.6	214.5	206.5	196.3	227.0	183.7

Standard errors (in parentheses) are bias-corrected and accelerated (BCa) bootstrap standard errors based on 1,000 replications.
# indicates a statistically significant male–female difference based on pooled OLS models with gender interactions (p<0.05).
*** p<0.001, ** p<0.01, * p<0.05, + p<0.1, b: significant after Bonferroni correction, f: significant after FDR correction
Notes: The following variables are controlled in the models: number of children ever born, health lifestyles in 2016, longevity PGS, social support in 2014, educational attainment, parental education, total of all assets in 2016, retirement status in 2016, chronological age at 2016, cohort, family size in 2016, number of living siblings in 2016, religious affiliation, and population stratification.
